# Supplementary material for: Condensation and asymmetric amplification of chirality in achiral molecules adsorbed on an achiral surface
Source: Nat Commun. 2023 Apr 13;14:2100. doi: 10.1038/s41467-023-37904-z (PMC10101975; doi:10.1038/s41467-023-37904-z)
Supplement: Supplementary file 1 — Supplementary Information [file 41467_2023_37904_MOESM1_ESM.pdf]

Supplementary Information for

Condensation and asymmetric amplification of chirality in achiral molecules adsorbed on an achiral surface

Huiru Liu[1,2#], Heping Li[3#], Yu He[1,2], Peng Cheng[1,2], Yi-Qi Zhang[1,2], Baojie Feng[1,2], Hui Li[3*], Kehui Wu[1,2,4*] and Lan Chen[1,2,4*]

[1]*Institute of Physics, Chinese Academy of Sciences, Beijing 100190, PR China*

[2]*School of physics, University of Chinese Academy of Sciences, Beijing 100049, PR China*

[3]*Beijing Advanced Innovation Center for Soft Matter Science and Engineering, Beijing University of Chemical Technology, Beijing 100029, PR China*

[4] *Songshan Lake Materials Laboratory, Dongguan, Guangdong, 523808, PR China*

*Email: lchen@iphy.ac.cn (L. C.), hli@buct.edu.cn (H.L), and khwu@iphy.ac.cn (K. W)

[#]These authors contributed equally to this work.

This PDF file includes:

Supplementary note 1. Lateral shift of heptamers

Supplementary note 2. Evolution of bias-dependent FFT images

Supplementary note 3. *Ab initio* Calculation of diffusion barriers

Supplementary note 4. Phase transition induced by opposite bias polarity

Supplementary note 5. Threshold voltage for phase transition

Supplementary note 6. Randomness of phase transition within multidomain boundary region

Supplementary note 7. Successive evolution of domain boundaries

Supplementary note 8. Evolution of the homochirality of CO cluster phase with domain B

Supplementary note 9. Nonlinear amplification of Frank model

Supplementary note 10. Evolution of domain boundaries triggered by tip pulse

Supplementary note 11. Comparison of different vdW-correction

Supplemenaty Figure 1 to 14

Supplementary Table 1. The calculated adsorption energy considering different Van der Waals types.

Supplemenatry references

Supplementary note 1. Lateral shift of heptamers

In our STM images, the two kinds of CO heptamers domains with different chirality (domain A and domain B) is identified as the area of heptamers with different orientations. Consequently, the domain boundaries are used to separate the area of heptamers with different orientations. To better show the position of domain boundaries, as an example, we superimpose the straight lines to reveal the orientations of the two neighbor domains in magnified upper part of Figure 1b, which is shown as Supplementary Fig. 1a.

Moreover, we can notice the long-range order of CO heptamers with same orientation is not very good in STM images. The lateral shifts of CO heptamers often occur, but they still keep the same orientation, which can be seen in the magnified central part of Figure 1b (see Supplementary Fig. 1b). In order to focus on the discussion of auto-amplification of enantiomeric excess, we regard the area of CO heptamers arrangements with same orientation as one kind of domain and ignore the lateral shifts of heptamers inside one domain.

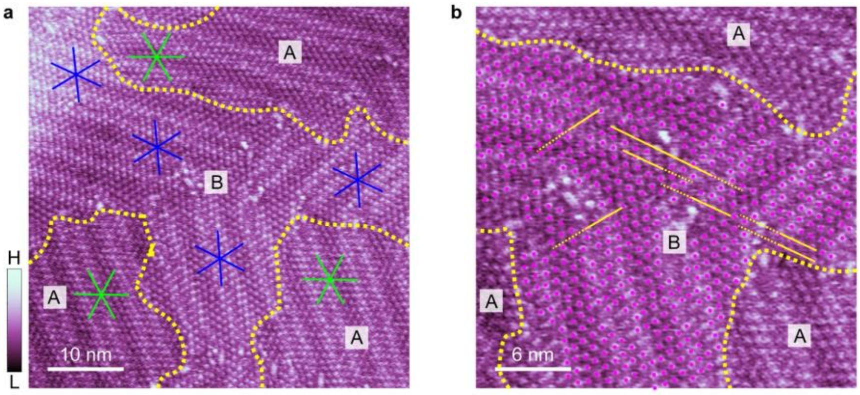

Supplementary Figure 1. Enlarged images of Figure 1b. a, Difference in orientations of domain A (green solid lines) and B (blue solid lines). Three equivalent directions of each domain are labeled by solid lines. Yellow dotted line marked the domain boundaries. **b,** Magnified central part of Figure 1b. Pink points represent the CO cluster from right-hand domain (domain B). The yellow straight lines mark the lattice shifts usually occur.

Supplementary note 2. Evolution of bias-dependent FFT images

Supplementary Figure 2 shows another group of FFT images at different bias, similar to Fig. 3 in the main tex. The STM images of the metastable uniform phase at high bias tends to have lower contrast due to lower diffusion barrier of CO monomers. However, even so, it’s clear for the CO monomer, as well as the refined structure of herringbone in the center of FFT images, suggesting without the loss of resolution of the tip under high bias.

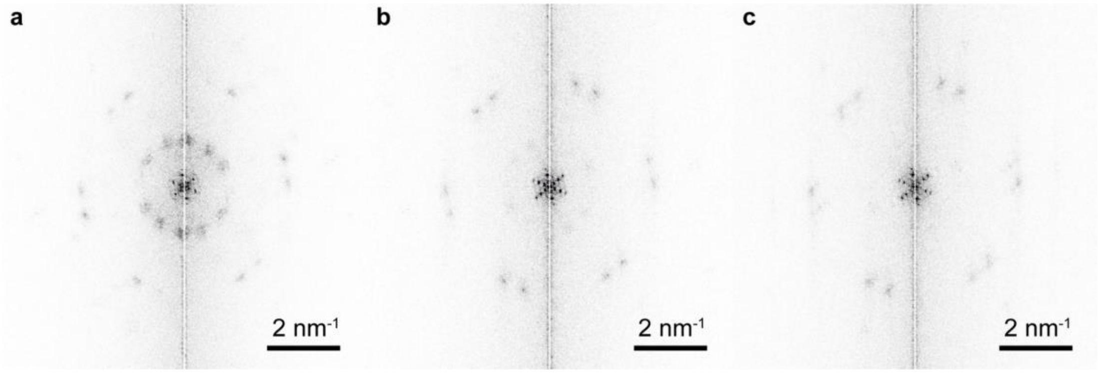

Supplementary Figure 2. The evolution of bias-dependent FFT images obtained from the STM images taken on the same area. Scanning parameter: V_{tip} = -0.1 V for **(a)**; 2.5 V for **(b)**; 3.0 V for **(c)** with the same current 5 pA.

Supplementary note 3. *Ab initio* Calculation of diffusion barriers

The diffusion barrier was estimated through scanning the potential energy surface (PES) of CO diffusing on the substrate. The relaxed PES scanning is a common method and widely used in study reaction mechanisms, studying bond stability, evaluating conformational flexibility, and locating transition structures especially for the surface diffusion problem [1-5]. For the structure at each scanning point, the x and y direction of carbon atoms was fixed, while the z direction of carbon atoms and the xyz directions of oxygen atoms were fully relaxed. The force criterion of structure optimization was 0.02 eV Å^{-1}.

Supplementary Figure 3 shows the diffusion barriers of CO in different sized cluster models. The estimated minimum barrier of diffusion of single CO molecule on Au(111) is approximately 0.055 eV (see Supplementary Fig. 3a). Such diffusion barrier can be remarkably increased with the increase of cluster size, which is attribute to the more intermolecular interaction pairs around an individual CO molecule. The diffusion barriers of CO in trimer and heptamer clusters are approximately 0.085 and 0.152 eV (Supplementary Fig. 3b and 3d), respectively. Such increase of the diffusion barrier should gradually become negligible in larger CO clusters, since the intermolecular Van de Waals interaction is a short-range interaction. Thus, the diffusion barrier of CO in phase transition should be approximately 0.1-0.2 eV, a smaller value than that of CO on other noble metal surface [6], indicating it's moderate for a tip volage to induce easier diffusion and phase transition of CO molecules on Au(111).

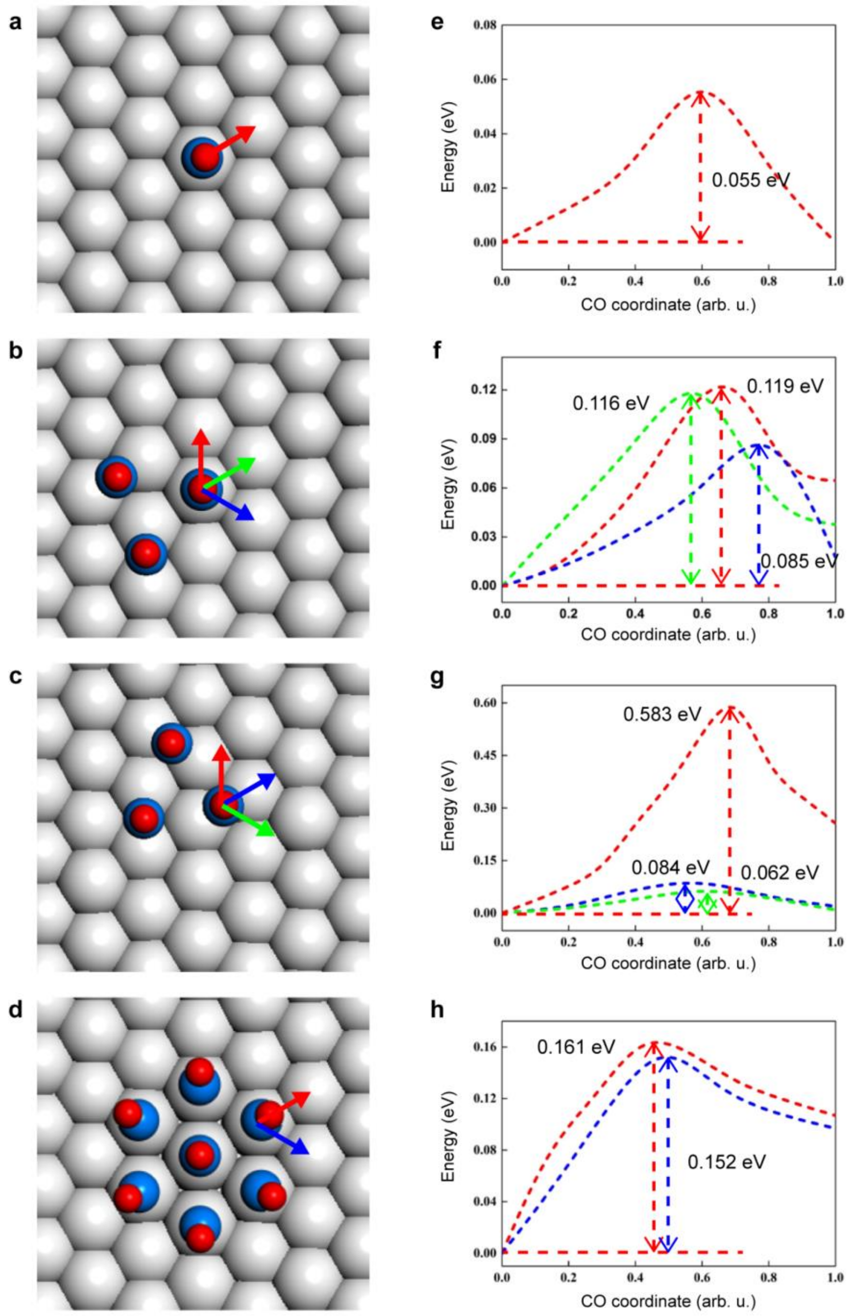

Supplementary Figure 3. The potential energy surface of CO diffusion on Au(111). The structural models (**a-d**) and corresponding diffusion barriers (**e-h**) for CO molecules on Au (111) surface. Red, blue, and grey balls represent oxygen, carbon, and gold atoms, respectively. Red, blue, and green arrows indicate the diffusion directions of the CO molecules, corresponding to the red, blue, and green curves in (**e-h**). The ordinate are relative energies, the energy of the initial structure is defined as “0”. The points on the curve represent the difference from the energy of the initial structure.

We further evaluate the barrier for the chirality transition process of the heptamer structure,

as shown in Supplementary Fig. 4. According to the structures of the left- and right-handed phases (Supplementary Fig. 4a and 4b), we can assume the transition path can be that the heptamer marked by the yellow circle is fixed and the heptamer marked by the red circle passes two Cu atoms of the substrate. As shown in Supplementary Fig. 4c, the cluster moving from site-a to site-c experiences the transition from the left-handed phase to the right-handed phase. The scanned potential energy surface along such phase transition path is shown in Supplementary Fig. 4d. The entire diffusion barrier of ≈ 1.39 eV, corresponding to the diffusion barrier of about 0.20 eV for each CO molecule. Such value is consistent to the diffusion barrier of single CO in heptamer, as shown in Supplementary Fig. 3h.

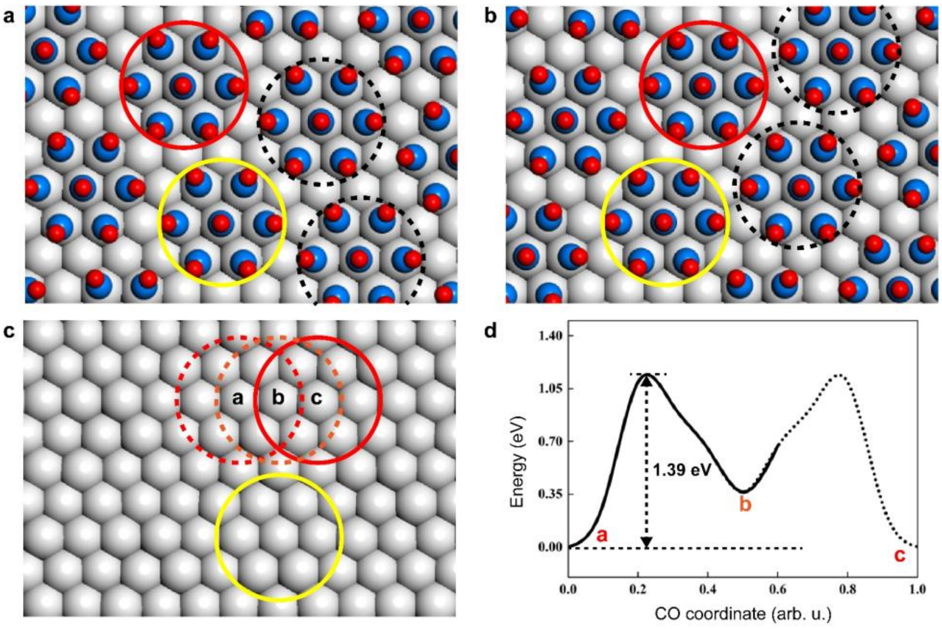

Supplementary Figure 4. Simulated phase transition model. a, b, Atomic models of the left- and right-handed cluster phases. **c,** Diagram simulating the phase transition process of a left-handed cluster to a right-handed cluster. The pathway of the red cluster moving from point-a to point-c on Au (111). **d,** Corresponding potential energy surface (PES) along the scanning path in **(c)**. The calculated potential barrier is about 1.39 eV. The ordinate represents relative energies, with the structural energy at point “a” as the reference energy, and all the points later on the curve are relative to this reference energy.

We further considered the energy barriers under the electric fields. Referring to the experimental electric field value (1.0 ~ 3.5 V nm^{-1}) in the positive tip bias, we have concluded the effect of the electric field into the DFT calculations, by setting E-fields of 0.10 V $Å^{-1}$ and 0.20 V $Å^{-1}$, respectively, as shown in Supplementary Fig. 5. We chose the diffusion modes of CO in trimer shown in Supplementary Fig. 5a to characterize the effect of the electric field on the barrier under the electric fields of E = 0.10 V $Å^{-1}$ (Supplementary Fig. 5c) and 0.20 V $Å^{-1}$ (Supplementary Fig. 5d), respectively. It is found that the applied electric field can apparently lower the diffusion barrier of CO. As shown in Supplementary Fig. 5d, with the electric field increased to 0.20 V $Å^{-1}$, the minimum barrier of CO in trimer is decreased from 0.085 to 0.058 eV. To further investigate the effect of electric field on CO diffusion, we selected the diffusion modes of CO in heptamer for the same calculation. Supplementary Figure 6 shows that, with the electric field increased from 0.00 to 0.20 V $Å^{-1}$, the minimum barrier of CO in heptamer is decreased from 0.152 to 0.138 eV. This result confirms that the STM tip at high bias can induce the molecular diffusion and facilitate the phase transition by lowering the diffusion barrier.

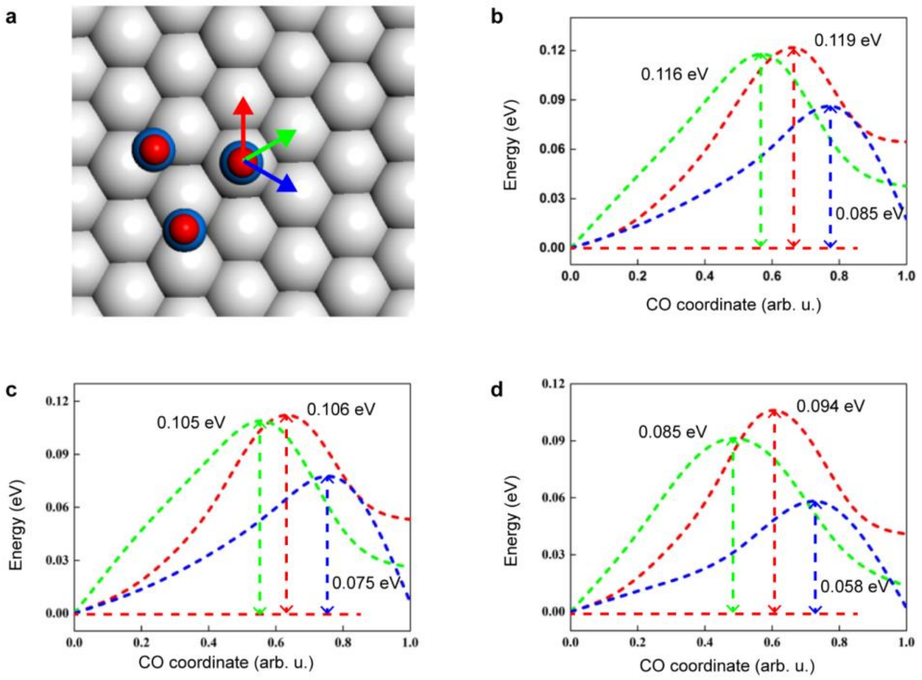

Supplementary Figure 5. Calculated diffusion barriers of CO in trimer on Au(111) surface. a, Structural models for estimating the diffusion barriers of CO molecules. Red, blue, and grey balls represent oxygen, carbon, and gold atoms, respectively. Red, blue, and green arrows indicate the diffusion directions of the CO molecules, corresponding to the red, blue, and green curves in (**b-d**).The DFT-calculated diffusion barriers without electric field **(b)**, with the electric field of 0.10 V $Å^{-1}$ **(c)**, and with the electric field of 0.20 V $Å^{-1}$ **(d)**, respectively. The ordinate are relative energies, the energy of the initial structure is defined as "0". The other data points on the curve represent the difference from the energy of the initial structure. The arrows marked the maximum diffusion barriers.

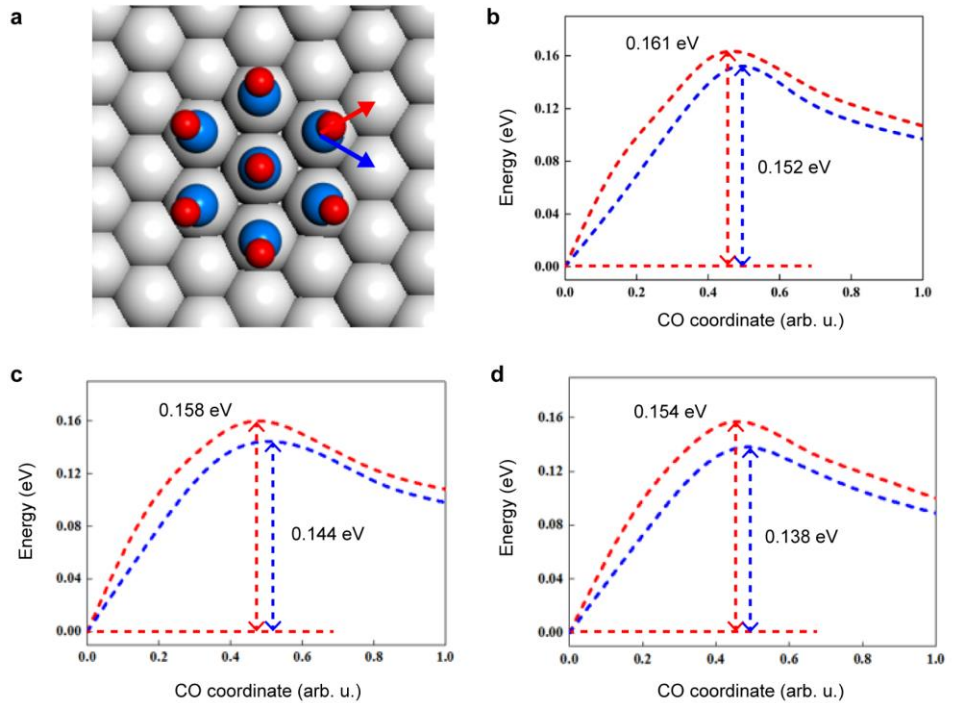

Supplementary Figure 6. Calculated diffusion barriers of CO in heptamer on Au(111) surface. a, Structural models for estimating the diffusion barriers of CO molecules. Red, blue, and grey balls represent oxygen, carbon, and gold atoms, respectively. The DFT-calculated diffusion barriers without electric field (**b**), with the electric field of 0.10 V Å^{-1} (**c**), and with the electric field of 0.20 V Å^{-1} (**d**), respectively. Red and blue curves correspond to the diffusion barriers of the CO molecules along the diffusion directions marked by the red and blue arrows in (**a**). The ordinate are relative energies, the energy of the initial structure is defined as "0". The other data points on the curve represent the difference from the energy of the initial structure. The arrows marked the maximum diffusion barriers.

Based on the above discussion, a quantitative dependence of the diffusion barriers on applied electric field can be obtained. We calculated the diffusion barriers of CO monomer, CO in trimer and CO in heptamer with the electric fields of 0.30 V Å^{-1}, 0.40 V Å^{-1} and 0.50 V Å^{-1}. Interestingly, we observed that the diffusion barriers of CO decrease linearly with the increase of electric field (Supplementary Fig. 7). This also confirms that the electric field can reduce the CO diffusion barrier and further promote the phase transition.

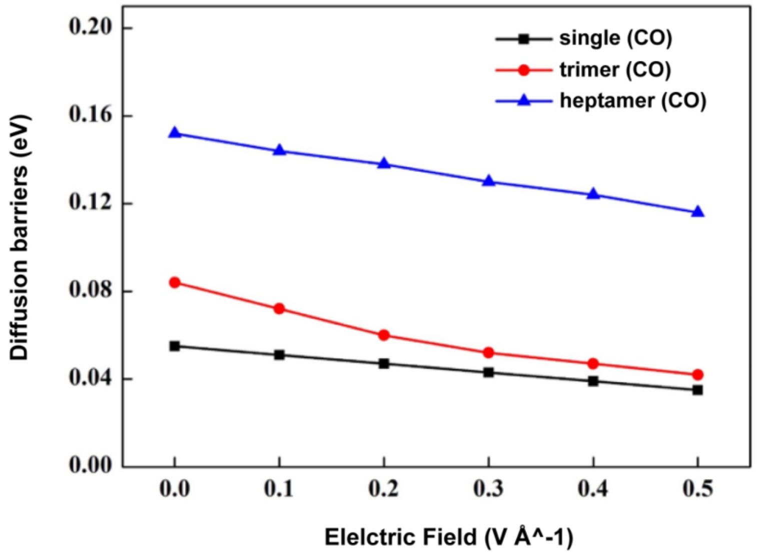

Supplementary Figure 7. Diffusion barriers as a function of applied electric field. Black, red and blue data points show the diffusion barrier as a function of the electric field for CO in monomer, trimer and heptamer, respectively**.**

Supplementary note 4. Phase transition induced by opposite bias polarity

Supplementary Figure 8 shows the phase transition induced after withdrawing a high negative tip bias, which is consistent with the behavior with the positive tip bias. It can be inferred that the chiral asymmetry amplification in our system can be stimulated by both polarities. But it should be emphasis that the formation and amplification of homochirality is not directly related to the bias voltage (neither electric field nor energy excitation). Just as reported before [7], asymmetric synthesis is not possible if system have reached complete thermodynamic equilibrium. The role of high bias voltage is to destroy the stable racemic structure (cluster phase) and make the system into metastable state (uniform phase), thus inducing the generation of small enantiomeric excess during recondensation process, resulting in chiral amplification.

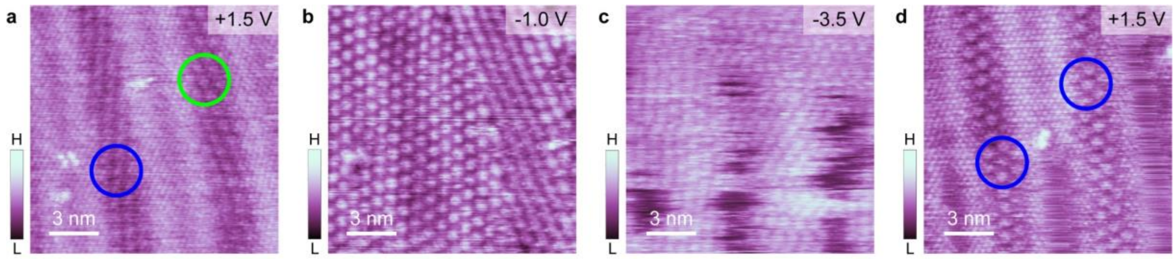

Supplementary Figure 8. Phase transition induced by an opposite bias polarity in the CO cluster phase on Au(111). a-d, A series of STM images similar to those in Fig. 4a-d but with tip biases of +1.5 V, -1.0 V, -3.5 V, and +1.5 V. The green and blue circles represent left-handed and right-handed domains. “H” and “L” above the color bar is the abbreviation of “High” and “Low”. Scanning currents are all 5.0 pA.

Supplementary note 5. Threshold voltage for phase transition

The threshold voltages for the transition of chiral cluster domain to a uniform phase varies as tunneling current is summarized in Supplementary Fig. 9. Red and black data point are from the threshold voltages under positive and negative tip bias respectively. And black solid line is fitted by linear function by formula $U \propto E \cdot (-\ln I)$. Each data point was checked repeatedly with about eight voltage numerical points, and error bar corresponds to about 0.15 eV. For the case of positive tip bias, the threshold voltage decreases as the tunneling current increases, indicating the transition from cluster phase to uniform phase is stimulated by electric field between tip and sample. It is because the direction of electric field is parallel to the dipole moment of CO molecule on Au(111). The fitted value of critical field is about 0.79 V Å^{-1}. Whereas, for the case of negative tip bias, corresponding to the direction of electric field is antiparallel to molecular dipole moment, the threshold voltage almost keeps same as tunneling current varies. We suppose the phase transition at negative bias is stimulated by electronic excitation or vibration excitation.

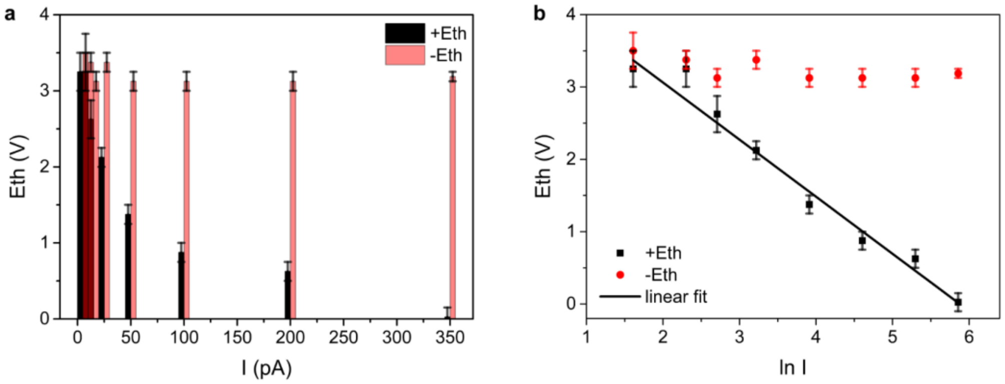

Supplementary Figure 9. The threshold voltage required in the phase transition from cluster phase to uniform phase. a, Histogram showing the dependence of E_{th} on tunneling current. **b,** By making the logarithm of the tunnelling current, linear relationship is fitted by formula $U \propto E \cdot (-\ln I)$. The discrete points come from the experimental statistics (around four independent repeated measurements per data point) and the solid lines are the corresponding linear fitting results. Error bars are defined by the half of the voltage difference between two adjacent phase transition, corresponding to about 0.15 V for all experimental data.

Supplementary note 6. Randomness of phase transition within multidomain boundary region

The conversion of CO uniform phase to the cluster phases from high to low bias voltage will choose chiral +13.9° and -13.9° orientations randomly, which always occurs. To better illustrate this issue, we show a group of STM images recording successive phases transition on a larger area (50×50 nm^2) of CO cluster phase with several small domains (see Supplementary Fig. 10), in which each image is scanned at low bias voltage after one-time scanning at high voltage. After operation of phase transition at first, the domain A and domain B occur randomly on the scanning area. In this process, the system still maintains the trend that as the operation times increase, the size of domain increases and the density of boundary decreases. Unlike the small

area in the main text (15×15 nm^2), more operation times are needed to achieve the homochirality on a larger area.

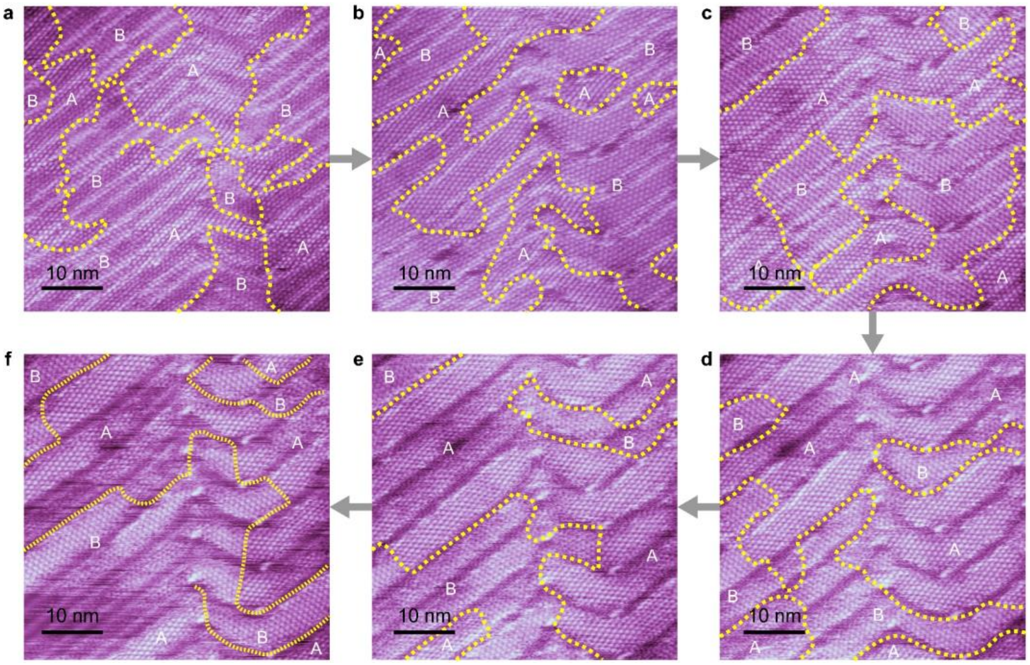

Supplementary Figure 10. The evolution of domains of CO cluster phase on Au(111) pattern with successive operation cycles at high voltage. a-f, Each STM image is scanned at low bias (-1.0 V, 6 pA) after one-time scanning at high bias (+3.5 V, 6 pA). Gray arrows represent the operation sequence. Yellow dotted lines marked the domain boundaries.

Supplementary note 7. Successive evolution of domain boundaries

In the process of the chiral amplification, the domain with homochirality can be grown by successive operation process at high bias voltage in a step-by-step way. As mentioned in the main text, domain boundaries tend to become less to minimize the system energy [8,9], furthering the chiral amplification of CO cluster phase. Supplementary Figure 11 contains a group of STM images recording successive operation of phase transition on a larger area of CO cluster phases, which showing a clear trend of domain boundaries, resulting in larger size of domain A.

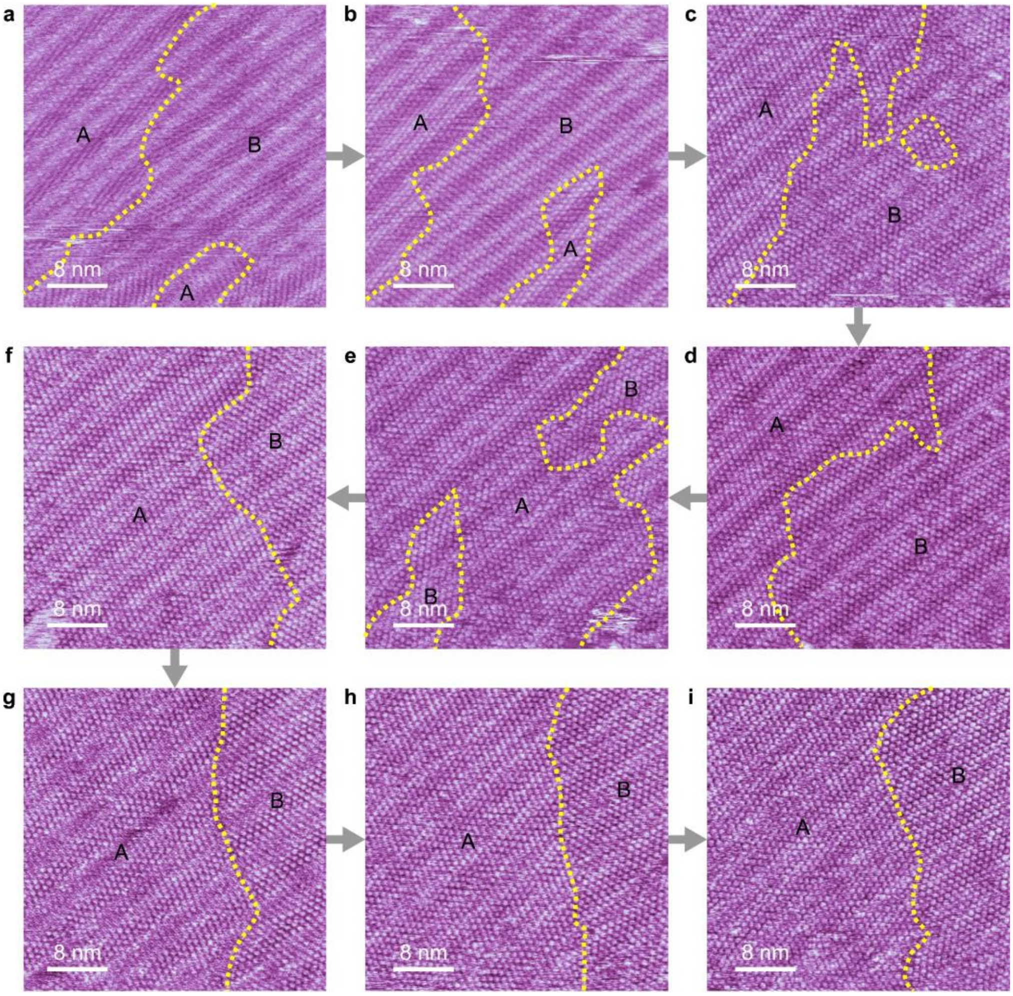

Supplementary Figure 11. The successive evolution of domain boundaries between two kinds of domains of CO cluster phase on Au(111). a-i, Each STM image is scanned at low bias (-0.1 V, 5 pA) after one-time scanning at high bias (-3.5 V, 5 pA). Gray arrows represent the operation sequence. Yellow dotted lines marked the domain boundaries.

Supplementary note 8. Evolution of the homochirality of CO cluster phase with domain B

We speculate that the chiral amplification here is driven by the domain boundary free energy under statistic fluctuation. Therefore, for the initial racemic condition, the formation probability of homochirality of left- and right-handed cluster phase should be equal. For example, Supplementary Figure 12 shows the generation of almost homochirality for right-

hand as a supplement to the Fig. 5 in the main text. Supplementary Figure 12 contains a group of STM images recording successive operation of phase transition on a larger area (40×40 nm^2) of CO cluster phases. It is obvious that the boundaries between domain A and B gradually become shorter and straighter, and the size of domain B with right-handed chirality became larger and cover the whole area finally, reflecting the chiral amplification.

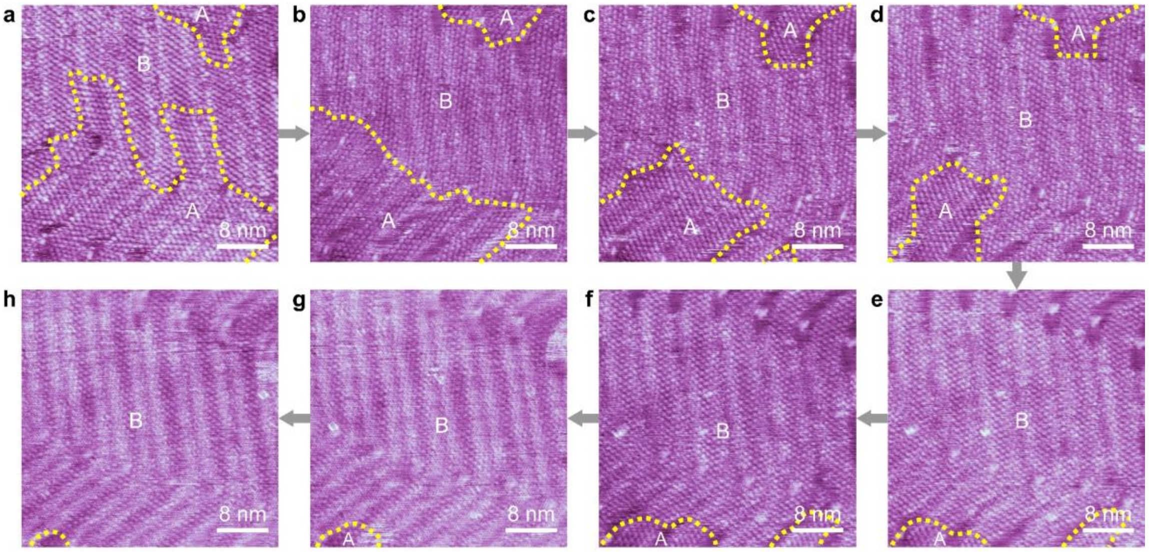

Supplementary Figure 12. The evolution of the homochirality of CO cluster phase with domain B on Au(111). a-h, Each STM image is scanned at low bias (-1.0 V, 5 pA) after scanning at a positive high bias. Gray arrows represent the operation sequence. Yellow dotted lines marked the domain boundaries.

Supplementary note 9. Nonlinear amplification of Frank model

The chiral amplification phenomenon in our work is more likely a "soldier and sergeants" process driven by interface energy that can be described by the modified Frank model. Supplementary Figure 13 shows the simulated P_D (ee) curve (Eqs. (5)) based on modified Frank model described in the main text. We choose rate constant $k_1 = k_2 = 0.5$, coefficient $\alpha = 21.48$, concentration $N_D = 1.1\ ^{10}$. As previous report, there is probability of the major or minority to form homochiral states determining by the randomness of statistical fluctuation.

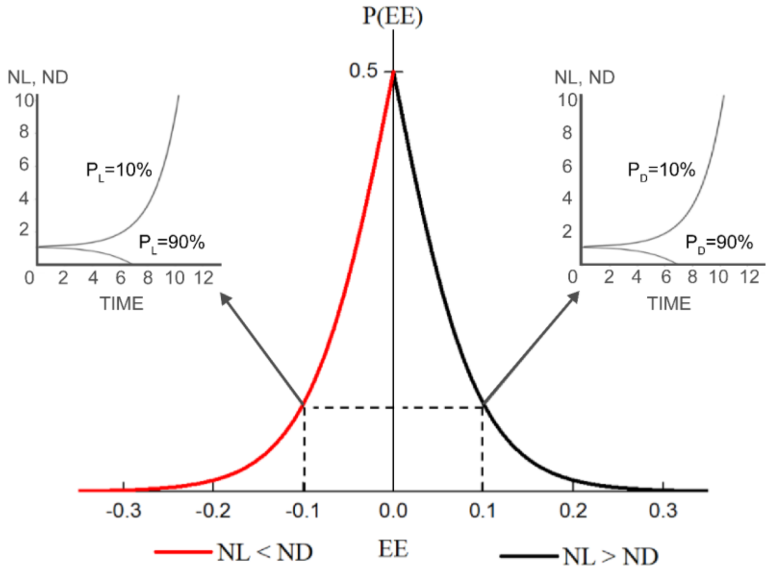

Supplementary Figure 13. The process of chiral amplification under stochastic Frank model. Solid curves are P_D (ee) curve with the red (or black) line corresponding to D concentration more (or less) than L concentration. The inset show the evolution of ee over time with the initial ee equal to ±10%.

Supplementary note 10. Evolution of domain boundaries triggered by tip pulse

In main text, the high bias voltage is applied through scanning the area of CO adlayer. In fact, we also tried to change the chirality of a few clusters near the boundaries of a racemic domain by applying voltage pulse. Supplementary Figure 14 shows a group of STM images which are obtained by successive scanning a same area at low bias voltage after several pulses of high bias voltage are applied. We can notice the chirality of clusters at the position of pulsing is changed, resulting in the domain boundary shorter. After several operation cycles of pulsing, almost the whole area covered by one domain with homochirality, suggesting the chirality amplification.

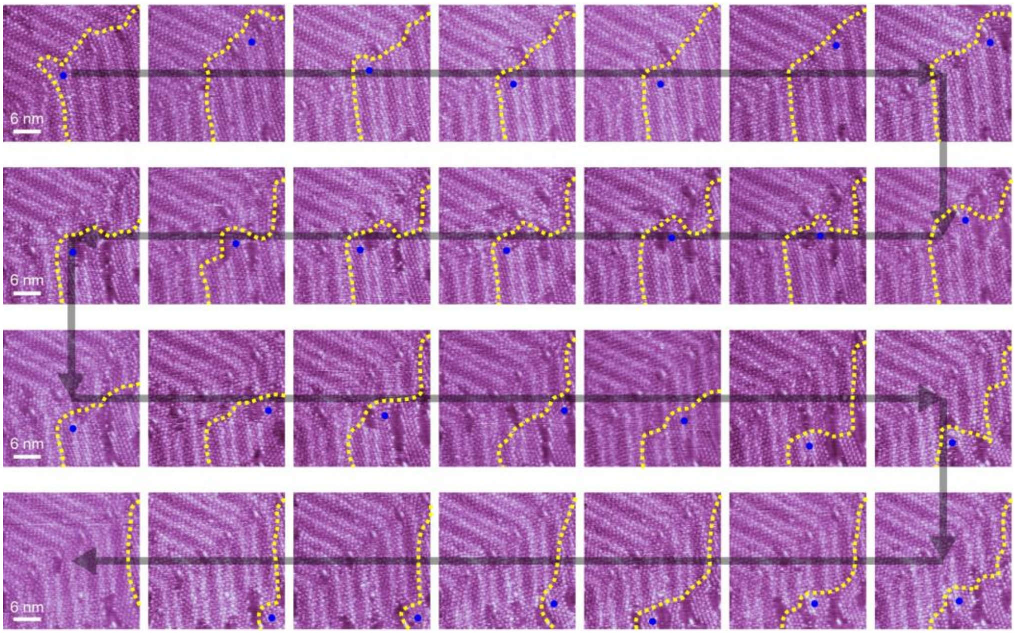

Supplementary Figure 14. The evolution of domain boundaries after operation processes by bias pulsing. Each image is scanned at the low bias (-50 mV, 5 pA). The blue circles mark the tip position of a pulse with 2.50±0.25 V and 1s duration. Gray arrows represent the operation sequence. Yellow dotted lines marked the domain boundaries. All images correspond to the same area with the size of 30×30 nm^2.

Supplementary note 11. Comparison of different vdW-correction

We have compared the adsorption energies without and with various dispersion correction functions (PBE [11], PBE-D2 [12], PBE-D3 [13,14], and optB86b [15]), as listed in Supplementary Table 1. It can be seen that PBE without any Van de Waals (vdW) correction significantly underestimate the adsorption energy of CO. Therefore, the vdW-correction is necessary for the CO adsorption calculation. On the other hand, PBE-D2, PBE-D3, and optB86b give very consistent adsorption energy values (-0.4 ~ -0.5 eV), indicating that the mainstream dispersion correction methods are all suitable for the present systems. Here, we employed the most widely used PBE-D3 method as the dispersion correction function in the present work.

Supplementary Table 1. The calculated adsorption energy considering different Van der Waals types (unit: eV).

vdWs type	Structure model		E_{ads}/7CO	E_{ads}/CO
PBE	Cluster phase	Left-handed	-1.054	-0.151
		Right-handed	-1.060	-0.151
	Uniform phase	Left-rotated	-0.812	-0.116
		Right-rotated	-0.797	-0.114
optB86b	Cluster phase	Left-handed	-2.971	-0.424
		Right-handed	-2.973	-0.425
	Uniform phase	Left-rotated	-2.726	-0.389
		Right-rotated	-2.711	-0.387
PBE-D2	Cluster phase	Left-handed	-3.632	-0.519
		Right-handed	-3.634	-0.519
	Uniform phase	Left-rotated	-3.537	-0.505
		Right-rotated	-3.562	-0.511
PBE-D3	Cluster phase	Left-handed	-3.150	-0.450
		Right-handed	-3.153	-0.450
	Uniform phase	Left-rotated	-2.838	-0.405
		Right-rotated	-2.826	-0.404

Supplementary references

1. Brust, D. J. & Gilbert, T. M. Density functional theory study of the d(10) series (H3P)(3)M(eta(1)-SO2) and (MenPh3-P-n)(3)M(eta(1)-SO_2) (M = Ni, Pd, Pt; N=0-3): SO_2 pyramidality and M-S bond dissociation energies. *Inorg. Chem.* **43,** 1116-1121 (2004).
2. Li, X., Chung, L. W., Paneth, P. & Morokuma, K. DFT and ONIOM (DFT: MM) Studies on Co−C bond cleavage and hydrogen transfer in B_{12}-dependent methylmalonyl-CoA mutase. Stepwise or concerted mechanism? *J. Am. Chem. Soc.* (2009).
3. Kozlowski, P. M., Kumar, M., Piecuch, P., Wei, L. & Jaworska, M. The cobalt-methyl bond dissociation in methylcobalamin: New benchmark analysis based on Density Functional Theory and completely renormalized coupled-cluster calculations. *J. Chem. Theory & Comput.* **8,** 1870 (2012).
4. Carrazana-García, J. A., Rodríguez-Otero, J. & Cabaleiro-Lago, E. M. A computational study of anion-modulated cation-π interactions. *J. Phys. Chem. B* **116,**

5860-5871 (2012).
5. Kwiecien, R. et al. Computational insights into the mechanism of radical generation in B_{12}-dependent methylmalonyl-CoA mutase. *J. Am. Chem. Soc.* **128,** 1287-1292 (2006).
6. Yang, K., Zhang, M. & Yu, Y. Effect of transition metal-doped Ni(211) for CO dissociation: Insights from DFT calculations. *Appl. Surf. Sci.* **399,** 255-264 (2017).
7. Absolute asymmetric synthesis under physical fields: Facts and fictions. *Chem. Rev.* **98,** 2391-2404 (1998).
8. Fasel, R., Parschau, M. & Ernst, K. H. Amplification of chirality in two-dimensional enantiomorphous lattices. *Nature* **439,** 449-452 (2006).
9. Hochberg, D. & Zorzano, M.-P. Reaction-noise induced homochirality. *Chem. Phys. Lett.* **431,** 185-189 (2006).
10. Silva-Dias, L. & Lopez-Castillo, A. Stochastic chiral symmetry breaking process besides the deterministic one. *Phys. Chem. Chem. Phys.* **19,** 29424-29428 (2017).
11. John et al. Generalized gradient approximation made simple. *Phys. Rev. Lett.* **77,** 3865 (1997).
12. Grimme, S. Semiempirical GGA-type density functional constructed with a long-range dispersion correction. *J. Comput. Chem.* **27,** 1787-1799 (2006).
13. Grimme, S., Antony, J., Ehrlich, S. & Krieg, H. A consistent and accurate ab initio parametrization of density functional dispersion correction (DFT-D) for the 94 elements H-Pu. *J. Chem. Phys.* **132,** 154104 (2010).
14. Grimme, S., Ehrlich, S. & Goerigk, L. Effect of the damping function in dispersion corrected density functional theory. *J. Comput. Chem.* **32,** 1456-1465 (2011).
15. Klime, J., Bowler, D. R. & Michaelides, A. A critical assessment of theoretical methods for finding reaction pathways and transition states of surface processes. *J. Phys.: Condens. Matter* **22,** 074203 (2010).
